# Leveraging the potential of social media: Unveiling the influence of customer-generated photos on customer behavior

**DOI:** 10.1371/journal.pone.0330201

**Published:** 2025-09-03

**Authors:** Asif Ali Safeer, Muhammad Abrar, Yewang Zhou

**Affiliations:** 1 Business School, Huanggang Normal University, Huanggang, China; 2 Lyallpur Business School, Government College University, Faisalabad, Pakistan; Nong Lam University, VIET NAM

## Abstract

Recent research shows that social media has enormous potential for customers and enterprises, but this potential has been largely untapped. This study investigates the influence of customer-generated photos on social media to drive customer visit intentions via the argument quality of online reviews, including perceived informativeness and persuasiveness, by integrating the direct and moderating effects of brand social media visual communication and controlling customer demographics such as gender, age, and income in the context of restaurants. This study collected 1,137 responses from different customers through an online survey. After carefully filtering data, 980 customer responses were analyzed using structural equation modeling. This study discovered that customer-generated photos significantly influenced perceived informativeness, persuasiveness, and customer visit intentions. Likewise, perceived informativeness and persuasiveness directly and indirectly contributed to increased customer visit intentions. In addition, brand social media visual communication directly influences to drive customer visit intentions toward restaurants. This study offers fresh insights for restaurant marketers to devise innovative marketing strategies to influence customer behavior toward eateries. Employing a social media ecosystem, this study contributes to the heuristic-systematic and elaboration likelihood models by examining consumer behavior in the hospitality industry.

## 1. Introduction

In recent years, individuals have become increasingly enthusiastic about capturing and disseminating photos via social media networks [1]. Social media networks have experienced an unprecedented surge in consumer-generated content, resulting in a significant shift in social media marketing communication [2]. The ubiquity of smart mobile devices has precipitated a notable proliferation in user-generated photos (UGPs). This explosion in visual content has become fundamental to engaging customers in sharing their activities, as seen by the striking number of over 576 million travel-related photographs shared on Instagram [3]. Photographs provide a spectrum of benefits transcending textual narratives, including informational depth, aesthetic appeal, and opportunities for self-expression [4]. Integrating visual elements into online reviews improves customer reviews’ authenticity and perceptual impact. Likewise, reviews incorporating visual content (i.e., photos) are considered more valuable and assist customers in making better decisions [5,6]. Humans have a distinct edge when processing visual information over text content; visuals are intuitively communicated to the brain and require fewer cognitive resources to retain [7]. Approximately two-thirds of the brain’s capacity is dedicated to visual processing, enabling quick assimilation of visual information at a rate about 60,000 times faster than textual information [1]. Scholars have begun to acknowledge the value of visual stimuli and urge more research based on photo data [1,6,8].

Social media has enormous potential today for the marketing of various products and services [9–11]. In April 2025, social media users reached 5.31 billion, representing 64.7% of the world population. Social media users grow by 7.6 new users per second, or 4.7% per year, with “adult” users comprising 87.3% of the world’s adult population [12]. The consequences of social media remain underleveraged. This study offers several novel contributions to strengthen theory and guides marketers in developing effective marketing strategies.

First, this study delves into examining the impact of customer-generated photos (CGPs) on the argument quality of online reviews, such as perceived informativeness and persuasiveness (PI and PP), and customer visit intentions to the restaurant. Previous studies are mainly based on secondary data, demonstrating that UGPs can improve customer engagement [8], online review helpfulness [5,7,13], usefulness [14], and consumer emotions [15], as prior research is mainly based on secondary data to examine UGPs [3,7,8,14,16]. There is a paucity of empirical evidence about the influence of CGPs in the hospitality industry. Empirical research is based on data collected from real customers, and the findings are grounded in real-world information, which may empower hospitality managers to make more effective decisions [1]. This study strives to add a new body of knowledge to theory and practice.

Second, this study examines the direct and indirect effects of argument quality of online reviews, including perceived informativeness (PI) and perceived persuasiveness (PP), on customer visit intentions. The argument quality of online reviews consists of PI and PP [17]. “Perceived informativeness refers to consumers’ overall perceptions regarding the information quality-related characteristics of online reviews, while perceived persuasiveness represents the general perceptions regarding the strength of persuasiveness embedded in online reviews” [17, p. 81]. Research indicates that 99.9% of customers consult online reviews to get information before purchasing [8]. Informative online reviews serve as primary sources of product information for many customers, facilitating the shaping of customer perceptions and persuasions, which guide them in effective purchase decisions [18]. Informative online reviews hold significant importance for businesses, while effective online reviews can influence customers’ persuasiveness [6]. Previous research demonstrates that the argument quality of online reviews can affect consumer purchase decisions [17,18]. However, studies examining the components of the argument quality of online reviews, including PI and PP on customer visit intentions to the restaurants, are rare. On the other side, although previous research highlighted the mediating role of perceived informativeness between conflicting consumer reviews and perceived correct purchase [18] but prior research overlooked the mediating role of the components of the argument quality of online reviews, including PI and PP, particularly between CGPs and customer visit intentions in the hospitality industry. This study embeds the direct and indirect (mediating) effects of perceived informativeness and persuasiveness to enhance theoretical and managerial contributions.

Third, this study proposes to incorporate the direct and moderating effects of brand social media visual communication on customer visit intentions to restaurants. Recent statistics indicate that global spending on social media advertising is expected to rise by USD 111.6 billion from 2024 to 2029, indicating a 47.66% growth [19]. Social media communication strategies enable companies to enhance their positioning and boost revenues [2]. For example, many well-known global companies, such as Dove, Apple, Nike, Pepsi, and Amazon, have successfully launched social media campaigns to improve customer perceptions. Previous studies mainly emphasized brand social media communications in a general or textual context, revealing that social media communication assists companies in strengthening positioning clarity, brand authenticity, brand evangelism, and consumer online impulse buying behavior [20–22]. There is dearth of research emphasizing the role of brand social media visual communication in the hospitality industry.

Finally, this study aims to control the confounding effects of customer demographics, such as gender, age, and income. Previous research supports the argument that user demographics may influence the use of social media [23]. Likewise, research demonstrates that consumer demographics, including gender, age, and income, may influence consumer purchase intentions for green products in the context of social media [24]. This study integrates the controlling effects of customer demographics, such as gender, age, and income, which may influence customer visit intentions to the restaurants. Based on the above potential research gaps, it is imperative to answer the following questions:

1) How do CGPs influence the component of the argument quality of online reviews, such as perceived informativeness and persuasiveness, and customer visit intentions to the restaurants?
2) To what extent do the direct and indirect impacts of argument quality of online reviews, such as PI and PP, influence customer visit intentions?
3) To what extent do the direct and moderating impacts of brand social media visual communication influence customer visit intentions?

With the support of potential research gaps, this study develops a comprehensive theoretical model via the lens of heuristic-systematic and elaboration likelihood models. The data were collected from real customers, and analysis was carried out using PLS-SEM. This study adds value to the literature by providing fresh theoretical and managerial insights.

## 2. Theoretical model and hypotheses development

**Fig 1:**
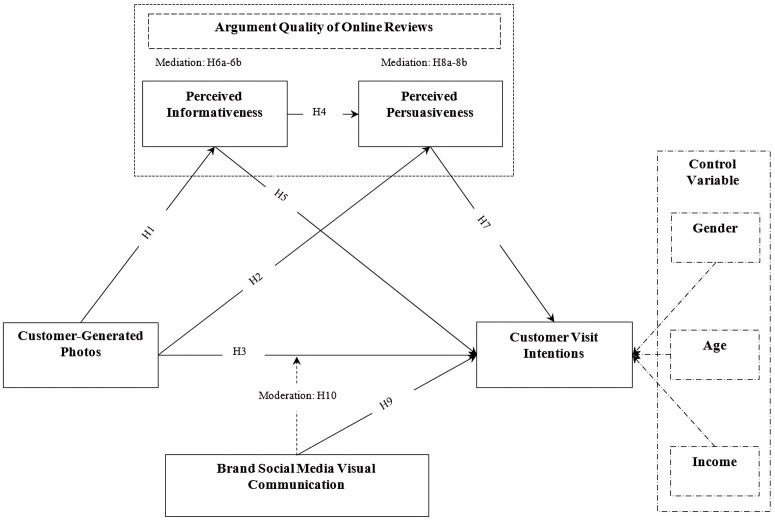
Theoretical model “Source: Authors own work”.

This study posits that customer-generated photos (CGPs) may positively influence customer visit intentions (CVI) via the argument quality of online reviews, including perceived informativeness (PI) and perceived persuasiveness (PP), by incorporating the direct and moderating effects of brand social media visual communication on CVI (see Fig 1) and integrating the controlling effects of customer demographics, including gender, age, and income. This framework is supported by the Heuristic-Systematic Model (HSM) and the Elaboration Likelihood Model (ELM).

### 2.1. Heuristic-systematic model (HSM)

The HSM plays a vital role in management information systems and hospitality literature [14,17,25]. HSM is a psychological framework that explains how people process information and make decisions [17,25]. It posits two distinct ways to persuasion (i.e., heuristic and systematic): Heuristic information processing facilitates quick assessments of stimuli by leveraging minimal information cues, whereas systematic information processing requires cautious, detailed, and intentional evaluations of issue-relevant cues [25]. Scholars have contended that photos provide supplementary informational cues, motivate consumers, and expedite their decision-making processes [1,17]. The HSM asserts that individuals are inclined to engage in systematic information processing when they have adequate motivation, cognitive capacity, and resources [26]. It implies that individuals possessing a high level of motivation are prone to critically assess the claims offered in a communication. Thus, high-quality arguments (i.e., PI and PP) are poised to exert a more pronounced impact on the individual’s decision-making processes [17].

### 2.2. Elaboration likelihood model (ELM)

The ELM is based on the two routes (i.e., the central and peripheral) of persuasion, which affect the likelihood of cognitive processes being used in interpreting a message [27]. ELM suggests that elaboration likelihood is low or high, depending upon an individual’s ability and motivation, which may determine the route and persuade an individual [28]. When customers have great motivation and are psychologically competent, they use the central route, involving profound argument quality of the message, such as its clarity, logic, and relevance, to shape customers’ attitudes and intentions. Conversely, when customers have low motivation or mental ability, they use the peripheral route, which often depends upon heuristic messages like visual attractiveness or source credibility [27,29].

### 2.3. Influence of customer-generated photos on argument quality of online reviews (i.e., perceived informativeness and persuasiveness) and customer visit intentions

Previous research mainly emphasized user-generated content (UGC) or consumer-generated content (CGC) in a general context, indicating that users or consumers create content on social media [2,30]. However, research centered around customer-generated photos (CGPs) is still limited. A recent literature review in Tourism Management demonstrates that photos are a more efficient type of content shared by customers on social media, which may provide new insights into both theory and practice [1]. Photos convey information distinctively than words; photos have more informative value and captivating power [14]. On the other hand, argument quality refers to “the strength or plausibility of persuasive argumentation” [31, p. 325]. Argument quality is a multifaceted concept that includes perceived informativeness and persuasiveness [17]. Previous research has asserted a positive correlation between the quality of photos and the informativeness and usefulness of online reviews [6,14]. The HSM posits that heuristic processing mechanisms play a pivotal role in facilitating individuals’ acquiring nuanced information from photos while expending minimal cognitive effort [17]. Consequently, CGPs serve as catalysts, motivating other customers to provide positive online reviews across various social media platforms, thereby augmenting the overall quality of arguments offered in online reviews. In addition, ELM suggests that when customers are involved in high-level decision making, they use the central route and rely on the cognitive value of the image, which provides them with important information [32]. It is hypothesized:

H1: CGPs are significantly associated with perceived informativeness.

The term “perceived persuasiveness” encapsulates the general perceptions concerning the effectiveness of persuasiveness inherent in online reviews [17]. Visual stimuli are more effective than textual information in capturing internet users’ attention and enhancing engagement [6]. This tendency is attributed to photos’ broad spectrum of objective information cues conferring higher persuasiveness than text. Therefore, assessments that include UGPs may be more persuasive than those that rely exclusively on descriptive text [6]. The HSM model demonstrates that heuristic processing enables quick stimulus evaluations by exploiting a few informational cues [17]. This study postulates that CGPs are heuristic cues that positively affect perceived persuasiveness, enhancing the argument quality of online reviews. From an ELM perspective, some customers show low motivation to engage with information deeply; they use the peripheral route for information processing, which is based on affective signals, such as the aesthetic quality of visuals and emotional appeal [28]. As a result, CGPs can be an effective persuasive trigger to influence customer behavior. It is hypothesized:

H2 CGPs are significantly associated with perceived persuasiveness.

UGP is a very effective means of communication that helps firms favorably impact customer behavior [8]. Prior research has employed various analytical approaches to investigate customer behavior. For example, researchers have used fuzzy-set qualitative comparative analysis and discovered significant associations between visual content and consumer decision-making processes [6]. Likewise, an experiment using Yelp zero-inflated Poisson regression analysis on 1,114 online reviews found that user-generated photos favorably influenced the perceived coolness, funniness, and helpfulness of reviews in the restaurant industry [33]. Many researchers have used deep learning approaches to examine the visual content of large datasets, including 35,356 UGPs from Flickr and 53,000 UGPs from TripAdvisor [34,35]. They discovered that UGP’s characteristics significantly influence tourists’ behaviors.

The HSM model explains how heuristic processing speeds up the evaluation of stimuli by effectively using minimum cognitive clues [17]. This study postulates that CGPs are heuristic cues that assist customers in using minimal cognitive resources and positively affect their intentions to visit the restaurant. On the other side, the ELM posits that individuals may use dual (the central and peripheral) routes to process information when they have a moderate level of motivation, which can influence their behavior [27,29]. As a result, customers can perceive CGPs cognitively and affectively, which may influence their visit intentions to the restaurant. It is hypothesized:

H3: CGPs are significantly associated with customer visit intentions.

### 2.3. Influence of perceived informativeness on perceived persuasiveness and customer visit intentions

In recent years, online review platforms have grown more sophisticated, allowing customers to attach photos with their distinctive reviews. As customers and companies acclimate to this burgeoning trend of visual content sharing, it is imperative to enhance knowledge of the influence of UGP online reviews [7]. Online reviews are highly effective in influencing customer decision-making processes. For example, 65% of customers consult online reviews when evaluating local cafes and restaurants, whereas 78% believe online evaluations have the same persuasive power and trust as recommendations from family or friends [6]. Online reviews are a valuable source of information and references for making purchase decisions. These reviews increase customer awareness and persuasiveness and significantly influence customer behavior [36]. The argument quality of online review informativeness is a systematic factor that assists customers in evaluating precise information about online reviews and boosts their persuasiveness [17]. Thus, it is predicted that the argument quality of online review informativeness may positively impact perceived persuasiveness. It is hypothesized:

H4: Perceived informativeness is significantly associated with perceived persuasiveness.

The growing popularity of social media platforms enables the sharing of tourism and hospitality experiences, accentuating the importance of online customer reviews [6]. Consumer-generated media (CGM) has evolved as an essential online information source. Prior research has indicated that 82% of American customers rely on online reviews to help them make travel-related choices [37]. Similarly, consumer-generated content serves as a vital source of information for seeking anonymous feedback and assisting customers in their future decision-making processes [2]. The HSM suggests that the argument quality of online informativeness provides systematic information to customers, guides them, and influences their behavioral intentions [17]. This study postulates that perceived informativeness is a systematic information process that may positively affect customer visit intentions to the restaurant. It is posited:

H5: Perceived informativeness is significantly associated with customer visit intentions.

### 2.4. Mediating role of perceived informativeness

Perceived informativeness of online reviews encapsulates the comprehensive knowledge gleaned from their textual content [18]. The length, ratings, and number of product attributes covered in an individual customer review all influence its perceived informativeness [18,38]. Prior research revealed that visual content, such as UGPs, imparts information uniquely compared to text and has greater informational value [14], significantly affecting customer behaviors [34,35]. Researchers articulated that informative online reviews have a substantial persuasive effect, influencing others to recommend the product to family or friends [6]. Likewise, online reviews serve as a valuable source of information and reference for making educated customer decisions. Such reviews increase customer knowledge and persuasion, significantly influencing consumer behavior [17,36]. This study postulates that perceived informativeness provides systematic information to customers that can potentially improve the relationships between CGPs and customers’ perceived persuasiveness and intent to visit a restaurant. It is proposed:

H6a: Perceived informativeness significantly mediates the relationships between CGPs and perceived persuasiveness.

H6b: Perceived informativeness significantly mediates the relationships between CGPs and customer visit intentions.

### 2.5. Influence of perceived persuasiveness on customer visit intentions

Perceived persuasiveness relates to customers’ ability to determine the level of relevancy associated with online reviews and high-quality persuasive arguments, which increase customers’ likelihood of making good decisions [6]. Online reviews increase customer understanding and persuasiveness and have emerged as critical sources for influencing customer purchase decisions [36]. Previous studies have evaluated how a review’s persuasiveness can be influenced by the reviewer’s traits, such as innovativeness [39], number of followers [40], expertise, and purchase decisions [41]. There is a dearth of research examining the impacts of online review persuasiveness on customer visit intentions to restaurants. This study postulates that perceived persuasiveness is a systematic processing factor that can influence customer visit intentions to the restaurant. It is proposed:

H7: Perceived persuasiveness is significantly associated with customer visit intentions.

### 2.6. Mediating role of perceived persuasiveness

Online reviewers’ persuasion strategies rely on their own expressive themes, which may be persuasive to other customers [36]. Previous studies have elucidated the critical importance of CGM as a primary source of online information, capable of influencing consumer decisions wisely [37]. Customers actively seek unsolicited viewpoints from fellow customers by engaging with consumer-generated content, improving their future decisions [2]. The HSM posits that the persuasive impact is contingent upon the argument quality of online reviews, providing customers with a systematic information processing framework that significantly influences their behavioral intentions [17]. In this background, it can be hypothesized that perceived persuasiveness can positively mediate between perceived informativeness, CGPs, and customer visit intentions to the restaurant.

H8a: Perceived persuasiveness significantly mediates the relationships between perceived informativeness and customer visit intentions.

H8b: Perceived persuasiveness significantly mediates the relationships of CGPs and customer visit intentions.

### 2.7. Direct and moderating role of brand social media visual communication

Social media communication often involves customers’ interaction with a brand [20]. This study explains that social media visual communication enhances interactions with customers by disseminating, exchanging, accessing, and collaboratively generating the visual content of a particular brand or restaurant. Previous studies have demonstrated that social media communication improves brand attachment and brand love, brand evangelism, brand loyalty, customer purchase decisions, and impulsive purchase behavior [2,21,22,42]. Prior research used brand social media communication in a general context. Brand social media communication focusing on visual content has little representation in existing literature. Previous research rarely explored the effects of brand social media visual communication content on customer visit intentions to the restaurant. The HSM suggests that systematic information assists customers and influences their behavioral intentions [17]. This study posits that brand social media visual communication provides systematic information to satisfy customers’ motivations, which can favorably affect their intentions to visit the restaurant. In an ELM context, brand social media visual communication may serve as peripheral signals that can influence customer attitudes and behavioral intentions [27]. For example, visually consistent and excellent brand photos, such as delicious dishes, aesthetic interior, and elegant crockery may influence customer visit intentions. Therefore, it is postulated:

H9: Brand social media visual communication significantly affects customer visit intentions.

Modern technological advancements and social media platforms have profoundly shifted the patterns of firm-consumer relationships, specifically in the hospitality industry [21]. Therefore, social media communication is valuable for businesses to interact with customers and influence their attitudes and behaviors [2,43]. The HSM primarily relies on heuristic and systematic information processing to influence consumer behavioral intentions [17]. Heuristic processing involves making quick, intuitive decisions based on basic cues or mental shortcuts [25]. Customers often depend on heuristic cues in social media environments. For example, when customers see photos shared by other customers on social media platforms, they often use heuristic processing to judge the restaurant’s atmosphere, food quality, and overall experience. These photos act as social proof and influence perceptions of the brand [14]. Systematic processing entails carefully evaluating information to determine its relevance and reliability [17]. In brand social media visual communication, systematic processing transpires when customers critically assess the restaurant’s visual content, including impressive photos and quality that match their preferences and values. A brand’s social media communication has the power to shape consumer attitudes and intentions [43]. In line with the ELM, brand and customer-generated photos can enhance other customers’ motivations, and they may use the central and peripheral routes for deep information processing, influencing their attitudes and behaviors [27,32]. The central route can assist customers in getting more information and understanding photos deeply, while the peripheral route can influence them through photos’ attractiveness and source credibility [28]. As a result, customers are more likely to interact with a brand and consider visiting a restaurant if it’s social media visual communication and CGPs align with their tastes and expectations. Thus, it is predicted that the interactive effects of brand social media visual communication and CGPs will significantly affect customer intentions to visit the restaurant. It is proposed:

H10: Brand social media visual communication will significantly moderate and influence customer visit intentions.

## 3. Materials and methods

This study used an online survey methodology to gather data from diverse audiences that often watch customer cuisine photos of various restaurants shared on various social media groups. Previous research has elucidated notable lacunae in the existing literature, accentuating the necessity of refining empirical and methodological contributions concerning CGPs in the hospitality industry [1]. In response to this scholarly exigency, the present research aims to fill these gaps by significantly contributing to the hospitality sector.

### 3.1. Sampling and data collection strategies

This study employed an online survey methodology for data collection. A snowball sampling approach was utilized to accomplish this objective. This methodological technique recruits an initial group of target respondents, which then aids in identifying and recruiting more people via survey referrals [2]. Snowball sampling is an effective strategy in a social media environment, as many customers commonly use social media and are comfortable sharing the survey link with other customers. This technique is highly efficient for collecting larger samples [2,21,22]. Respondents were not restricted to specific cuisines or restaurant photos; they were independent in sharing their views based on photos viewed on social media platforms (i.e., Facebook and Instagram). The survey was launched on a famous survey platform (i.e., www.wjx.cn) from December 18, 2023, to March 16, 2024. The participants responded to the survey based on photos they had recently viewed on social media platforms and their most recent visit to the same restaurant. The survey responses were collected on a seven-point Likert scale. A large cohort of respondents were contacted using social media platforms like Facebook and Instagram to obtain feedback. After completing the online survey, participants were requested to share it with their acquaintances, including family, friends, and colleagues who satisfy the specified requirements.

This study involves human participation and was approved by the Ethical Review Committee (Ref. No. HGNU/ERC/23/0101) at the Business School, Huanggang Normal University, Huanggang, China. This study was conducted in accordance with the local legislation and institutional requirements. All participants provided written, informed, voluntary consent before participation. This study did not consider the minors for data collection. In addition, participants could withdraw their participation while completing the survey.

### 3.2. Measures

Empirical studies are generally based on Likert scales, depending on the specific study context. However, seven-point Likert scales offer a more nuanced understanding of the under-investigated constructs [44]. This study has employed seven-point refined Likert scales derived from previous studies. The scale for customer-generated photos was refined and assessed using three items [45]. The argument quality of online reviews, including perceived informativeness and persuasiveness, was evaluated with three and four items, respectively [6,17]. The brand social media visual communication scale was refined and assessed using four items [20], and customer visit intentions were appraised using three items [46].

### 3.3. Survey pretest

A survey pretest is an initial assessment conducted with a small sample of respondents to evaluate the efficacy and suitability of a survey instrument before its full-scale deployment. The primary goal of the pretest is to identify and rectify any potential survey issues, such as unclear questions or technical glitches [2,22]. By administering the online survey to a smaller subset of the target population, scholars can obtain valuable feedback that can be used to guide changes and improvements to the survey, increasing its reliability and validity before it is widely distributed. A preliminary analysis was performed on 95 responses to assess the reliability and validity of all questions. The results revealed that all loading values ranged from 0.78 to 0.96, Cronbach’s alpha and composite reliability were greater than 0.70, and the average variance extracted (AVE) was greater than 0.50, satisfying the required threshold [44]. Following that, an additional 1,137 responses were collected for this study. During the data screening process, 157 responses were excluded, and 980 responses were considered for analysis. Table 1 displays the respondents’ information.

**Table 1:** Respondents’ Information.

Description	Number	%
**Sample size**	**980**	100.00
**Gender**		
Male	520	53.06
Female	460	46.94
**Age**		
20–26	437	44.59
27–33	321	32.76
34–40	117	11.94
41–47	105	10.71
**Education**		
High School	34	3.47
Bachelor	482	49.18
Master	418	42.65
Other Professional Degree	46	4.69
**Occupation**		
Student	436	44.49
Private Firm Employee	391	39.90
Government employee	122	12.45
Entrepreneurs	31	3.16
**Monthly Family Income (PKR)**		
Up to 50,000	401	40.92
50,001- 100,000	321	32.76
100,001- 150,000	133	13.57
150,001- 200,000	42	4.29
More than 200,000	83	8.47
**Usage of social media**		
**Do you use social media platforms regularly (Facebook, Instagram, etc.)?**	**Yes**	

“Source: Authors own work”.

The sample size was calculated using the G*Power test via G*Power software version 3.1.9.7. We performed the G*Power test with two-tailed, the effect size (0.15) and power (0.95) at 0.05 significance level, based on the earlier research recommendations [47,48]. The findings revealed that a minimum sample size of 89 was required for this study. To ensure precise sample size, another criterion was applied: the minimum sample size must be ten times the maximum number of arrowheads directed at a latent variable within the PLS path model [44]. In this case, the sample size should be 170. In addition, a review of previous studies revealed that larger sample sizes (exceeding 800 or 900) yield more valuable insights [30,49]. Similarly, scholars recommended that larger sample sizes improve the accuracy and reliability of PLS-SEM estimations [44]. Therefore, a larger sample size (i.e., 980) was considered for this study.

## 4. Results

In contemporary hospitality research, partial least squares structural equation modelling (PLS-SEM) has become a ubiquitous analytical technique [2]. This study used the PLS-SEM technique to unveil novel insights. PLS-SEM is a very useful technique for examining complex models, particularly for determining mediation [44]. The theoretical model can be analyzed in measurement and structural model evaluation.

Before commencing the analysis, a critical preliminary step involves the screening of data to remove anomalous responses. Therefore, SPSS software (version 25) was used to identify outliers and biased straight responses, removing 157 responses. This study employed the kurtosis and skewness method to determine the data’s normality. Table 2 delineates that all kurtosis and skewness values (±1.96 at a significance level of 0.05) satisfied the specified threshold [50]. In addition, Table 2 displays descriptive statistics involving mean and standard deviation.

**Table 2:** Descriptive statistics, data normality, construct reliability, and validity.

Construct	Code	Loading	Mean	SD	KURT	SKW
**Customer-Generated Photos (α = 0.93, CR = 0.94, AVE = 0.82)**						
I am satisfied with the food photos shared by other customers on social media platforms about this restaurant.	CGPs1	0.88	4.40	1.60	−0.73	−0.28
The photos of the food shared by other customers on social media platforms about this restaurant meet my expectations.	CGPs2	0.85	4.36	1.53	−0.69	−0.30
The food photos shared by other customers on social media platforms about this restaurant are better than other restaurants.	CGPs3	0.97	4.36	1.46	−0.41	−0.29
**Argument Quality of Social Media Reviews**						
**Perceived Informativeness (α = 0.87, CR = 0.87, AVE = 0.70)**						
The online reviews on photos offer helpful information regarding the restaurant.	PI1	0.80	4.30	1.35	−0.34	−0.22
The online reviews on photos offer comprehensive information regarding the restaurant.	PI2	0.86	4.27	1.39	−0.49	−0.28
The online reviews on photos offer up-to-date information regarding the restaurant.	PI3	0.84	4.32	1.35	−0.35	−0.27
**Perceived Persuasiveness (α = 0.89, CR = 0.89, AVE = 0.67)**						
The photo arguments of online reviews are convincing.	PP1	0.81	4.34	1.30	0.11	−0.54
The photo arguments of online reviews are good.	PP2	0.75	4.47	1.34	−0.32	−0.50
The photo arguments of online reviews are persuasive.	PP3	0.83	4.44	1.40	0.09	−0.45
The photo arguments of online reviews are strong.	PP4	0.88	4.55	1.40	−0.10	−0.43
**Customer Visit Intentions (α = 0.90, CR = 0.90, AVE = 0.75)**						
This restaurant is one that I would like to visit.	CVI1	0.86	4.39	1.22	0.48	−0.51
I would definitely visit this restaurant.	CVI2	0.86	4.50	1.29	−0.02	−0.25
It is my pleasure to eat at this restaurant.	CVI3	0.87	4.56	1.21	0.62	−0.40
**Brand Social Media Visual Communication (α = 0.92, CR = 0.92, AVE = 0.75)**						
The social media visual communication of this restaurant brand fulfills my expectations.	BSMVC1	0.84	4.09	1.19	−0.10	−0.48
The social media visual communication of this restaurant brand is highly appealing.	BSMVC2	0.89	4.31	1.19	0.09	−0.60
I am pleased with the social media visual communication of this restaurant brand.	BSMVC3	0.86	4.12	1.16	0.39	−0.44
The social media visual communication of this restaurant brand is highly effective, particularly when compared to other restaurants.	BSMVC4	0.87	4.31	1.22	0.05	−0.50

Bias may occur in responses during the data collection process. As a result, this study used two widely accepted methods to detect common method bias (CMB). First, this study applied the variance inflation factor (VIF). The findings revealed that all VIF values were less than 5, indicating no CMB in the data [51]. Second, this study applied Harman’s single factor (HSF). The results revealed that the HSF value (48.41%) was less than 50%, demonstrating that the data is free from CMB [52].

### 4.1. Measurement model evaluation

The measurement model evaluation aims to ensure that the proposed model constructs effectively demonstrate reliability, convergent validity, and discriminant validity [44]. Table 2 shows that all indicators’ loading values were greater than 0.70, and convergent validity (AVE) values above 0.50, showing that the proposed model possesses construct reliability and convergent validity [53]. The discriminant validity was measured using the Heterotrait-Monotrait (HTMT) Ratio, a well-known method [54]. Table 3 proves that discriminant validity has been achieved using the HTMT method [54].

**Table 3:** Discriminant validity; Heterotrait-Monotrait Ratio.

Construct	1	2	3	4	5
**Brand Social Media Visual Communication**					
**Customer Visit Intentions**	0.57				
**Customer-Generated Photos**	0.69	0.55			
**Perceived Informativeness**	0.44	0.49	0.54		
**Perceived Persuasiveness**	0.56	0.52	0.54	0.51	

### 4.2. Structural model evaluation

The structural model evaluates multicollinearity, coefficient of determination (R²), model fit, predictive relevance (Q²), and the consequences of the relationships between constructs to draw meaningful conclusions [44]. This research evaluated the model’s multicollinearity using the variance inflation factor (VIF). The findings showed that all VIF values were less than 5, implying no multicollinearity and that the data is free from bias [44,53]. The R^2^ results suggest that the theoretical model has high explanatory power by explaining the variance of exogenous variables on endogenous variables, with R^2^ values of 0.29, 0.36, and 0.44 for perceived informativeness, perceived persuasiveness, and customer visit intentions I, respectively [55]. The model fit was assessed using SRMR metrics. The results showed that the SRMR value for the estimated model was 0.05 (saturated model 0.03) (i.e., < 0.08) and the NFI value was 0.92 (> 0.90), demonstrating an excellent model fit [53]. This study utilized a blindfolding metric to assess the model’s predictive relevance (Q²). The findings indicated the model’s satisfactory predictive relevance [44], with Q² values of 0.19, 0.23, and 0.31 for PI, PP, and CVI, respectively.

**Fig 2:**
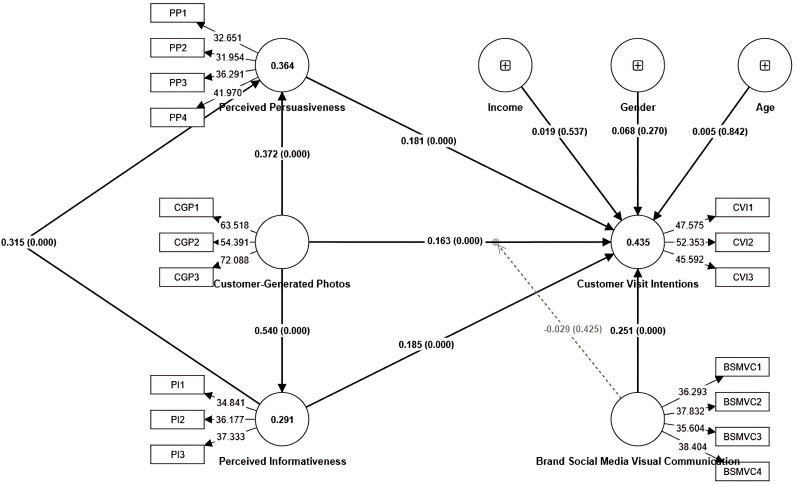
Structural Model Results “Source: Authors own work”.

The path analysis and proposed hypotheses were assessed using the bootstrap method with 10,000 sub-samples, two-tailed at a significance level of 0.05 [44,53]. The path analysis was performed to confirm the theoretical model’s results. Table 4 reveals the path analysis from Model 1 to Model 4, which validates the findings. After performing path analysis, the hypotheses were assessed. Table 5 and Fig 2 comprehensively demonstrate that customer-generated photos are strongly associated with perceived informativeness, perceived persuasiveness, and customer visit intentions, showing acceptance of H1-H3. The findings reveal that perceived informativeness is positively associated with customer visit intentions and perceived persuasiveness, indicating acceptance of hypotheses 4–5. Likewise, the results indicate that perceived persuasiveness is positively associated with customer visit intentions. Thus, H7 has been accepted. Moreover, the results show that brand social media visual communication is significantly associated with customer visit intentions, leading to H9 acceptance. The findings indicate that customer demographics, including gender, age, and income, had no impact on customer visit intentions.

**Table 4:** Path Analysis.

Relationship	Model-1	Model-2	Model-3	Model-4
	β value	β value	β value	β value
Customer-Generated Photos - > Customer Visit Intentions	0.55*** (s)	0.31*** (s)	0.31*** (s)	0.16*** (s)
Customer-Generated Photos - > Perceived Informativeness	________	0.54*** (s)	0.54*** (s)	0.54*** (s)
Customer-Generated Photos - > Perceived Persuasiveness	________	0.54*** (s)	0.37*** (s)	0.37*** (s)
Perceived Informativeness - > Customer Visit Intentions	________	0.19*** (s)	0.19*** (s)	0.19*** (s)
Perceived Persuasiveness - > Customer Visit Intentions	________	0.26*** (s)	0.26*** (s)	0.18*** (s)
Perceived Informativeness - > Perceived Persuasiveness	________	________	0.31*** (s)	0.31*** (s)
Brand Social Media Visual Communication - > Customer Visit Intentions	________	________	________	0.25*** (s)
Customer-Generated Photos - > Perceived Informativeness - > Customer Visit Intentions	________	0.10*** (s)	0.10*** (s)	0.10*** (s)
Perceived Informativeness - > Perceived Persuasiveness - > Customer Visit Intentions	________	0.14*** (s)	0.08*** (s)	0.06*** (s)
Customer-Generated Photos - > Perceived Informativeness - > Perceived Persuasiveness	________	________	0.17*** (s)	0.17*** (s)
Customer-Generated Photos - > Perceived Persuasiveness - > Customer Visit Intentions	________	________	0.10*** (s)	0.07*** (s)
Customer-Generated Photos - > Perceived Informativeness - > Perceived Persuasiveness - > Customer Visit Intentions	________	________	0.04** (s)	________
Brand Social Media Visual Communication x Customer-Generated Photos - > Customer Visit Intentions	________	________	________	−0.03 (ns)
Age - > Customer Visit Intentions	0.02 (ns)	0.01 (ns)	0.01 (ns)	0.00 (ns)
Gender - > Customer Visit Intentions	0.07 (ns)	0.05 (ns)	0.05 (ns)	0.07 (ns)
Income - > Customer Visit Intentions	0.02 (ns)	0.02 (ns)	0.02 (ns)	0.02 (ns)
	**R**^**2**^ **value**	**R**^**2**^ **value**	**R**^**2**^ **value**	**R**^**2**^ **value**
Customer Visit Intentions	0.31	0.40	0.40	0.43
Perceived Informativeness	________	0.29	0.29	0.29
Perceived Persuasiveness	________	0.29	0.36	0.36

Note: “(t >2.58 at **p <0.01); (t >3.29 at ***p <0.001); (two-tailed)”; (s) = significant; (ns) non-significant.

**Table 5:** Hypothesis Results.

Hyp.	Relationship	β value	CI 2.5%	CI 97.5%	t value	Mediation
**Direct Effects**						
H1	Customer-Generated Photos - > Perceived Informativeness	0.54*** (s)	0.48	0.60	18.54	
H2	Customer-Generated Photos - > Perceived Persuasiveness	0.37*** (s)	0.29	0.45	8.81	
H3	Customer-Generated Photos - > Customer Visit Intentions	0.16*** (s)	0.07	0.25	3.50	
H4	Perceived Informativeness - > Customer Visit Intentions	0.19*** (s)	0.11	0.27	4.41	
H5	Perceived Informativeness - > Perceived Persuasiveness	0.31*** (s)	0.23	0.40	7.32	
H7	Perceived Persuasiveness - > Customer Visit Intentions	0.18*** (s)	0.09	0.27	3.81	
H9	Brand Social Media Visual Communication - > Customer Visit Intentions	0.25*** (s)	0.16	0.34	5.57	
**Mediation Effects**						
H6a	Customer-Generated Photos - > Perceived Informativeness - > Perceived Persuasiveness	0.17*** (s)	0.12	0.22	6.35	Partial
H6b	Customer-Generated Photos - > Perceived Informativeness - > Customer Visit Intentions	0.10*** (s)	0.06	0.15	4.11	Partial
H8a	Customer-Generated Photos - > Perceived Persuasiveness - > Customer Visit Intentions	0.07*** (s)	0.03	0.11	3.43	Partial
H8b	Perceived Informativeness - > Perceived Persuasiveness - > Customer Visit Intentions	0.06*** (s)	0.03	0.09	3.41	Partial
**Moderation Effects**						
H10	Brand Social Media Visual Communication x Customer-Generated Photos - > Customer Visit Intentions	−0.03 (ns)	−0.10	0.04	0.80	
**Control Effects**						
Age - > Customer Visit Intentions		0.00 (ns)	−0.04	0.06	0.20	
Gender - > Customer Visit Intentions		0.07 (ns)	−0.05	0.19	1.10	
Income - > Customer Visit Intentions		0.02 (ns)	−0.04	0.08	0.62	

Note: “(t >3.29 at ***p <0.001); (two-tailed)”; (s) = significant; (ns) non-significant.

#### 4.2.1. Evaluation of mediation effects.

This research examined direct and indirect structural model relationships to evaluate the effects of mediation [44]. The results demonstrated that perceived informativeness partially mediated the associations between customer-generated photos and customer visit intentions, contributing to H6a-b acceptance. Similarly, perceived persuasiveness served as a decisive construct that partially mediated the associations between customer-generated photos and customer visit intentions, as well as between perceived informativeness and customer visit intentions, indicating the acceptance of H8a-b.

#### 4.2.2. Evaluation of moderation effects.

This study estimated the moderating impact by evaluating the interaction effects of brand social media visual communication and customer-generated photos on customer visit intentions as brand social media visual communication x customer-generated photos - > customer visit intentions (*β* = −0.03; *p* = 0.43). The results showed that the interaction between brand social media visual communication and customer-generated photos did not affect customer visit intentions. Therefore, H10 was rejected.

### 4.3. Importance performance map analysis (IPMA)

**Fig 3:**
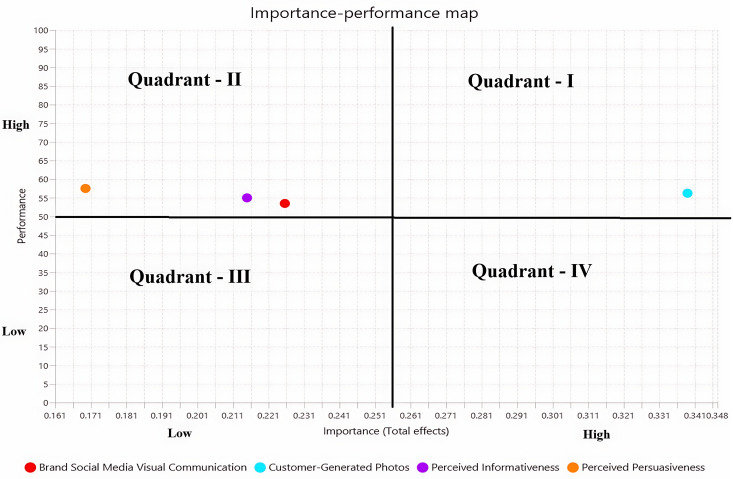
IPMA Results “Source: Authors own work”.

IPMA provides important guidelines for effective managerial actions that assist managers in making strategic decisions in the best interests of the stakeholders [56]. IPMA is based on a two-component map (i.e., importance on the X axis and performance on the Y axis), which indicates the exogenous constructs’ effects on the endogenous constructs. These effects highlight the importance and performance of the proposed constructs [44]. Fig 3 reveals that the component of the argument quality of online reviews, such as perceived informativeness and persuasiveness, as well as brand social media visual communication, have high performance scores but low importance scores. On the other hand, customer-generated photos have both high scores in terms of importance and performance. Therefore, companies should strive to put their attention from moving quadrant-II to quadrant-I to cultivate overall high business performance.

## 5. Discussions

The findings provide compelling insights and effectively contribute to theories and literature. First, the findings imply that customer-generated photos (CGPs) significantly influenced the perceived argument quality of online reviews, making them more informative and persuasive. CGPs act as heuristic cues that convey useful information, influence customers’ perceptions and intentions, and demonstrate the persuasive power of visual content on social media platforms [6,14]. The heuristic processing theory suggests that individuals often rely on simple cues, such as visual stimuli, to make quick judgments [25]. CGPs serve as tangible evidence of the dining experience, providing potential customers with visual cues about the restaurant’s food, ambience, and overall atmosphere. On the other hand, customers perceive social media photos depending on their motivation. For example, highly motivated customers use central routes for deep information processing to get more informed. In contrast, low-motivated customers use peripheral routes to review the photo quality and source credibility for their persuasions [27,29]. Second, the positive impact of CGPs on customer intentions to visit the restaurant aligns with the HSM principles, which explain how individuals process information and make decisions in persuasive communication contexts [17]. Likewise, social media may motivate customers via central and peripheral routes to aid them in their decision making [28].

Third, it was discovered that the argument quality of online reviews, such as perceived informativeness, significantly influenced the perceived persuasiveness of online reviews and customer visit intentions to the restaurant. This suggests that, beyond merely providing information, the quality of the arguments presented in online reviews plays a decisive role in persuading and shaping consumers’ perceptions and behavioral intentions in the restaurant context. The findings of this study align with previous research, indicating that consumer-generated content (i.e., general or textual) provides valuable information that influences consumer purchase decisions in the hospitality and tourism industry [2,37]. Fourth, this study revealed a significant relationship between the perceived persuasiveness of online reviews and customer visit intentions to the restaurant. The finding accentuates the importance of online reviews’ content and persuasiveness in shaping restaurant industry consumer behavior. Customers prefer to decide on dining selections based on the arguments offered in reviews, with persuasive evaluations affecting their decision to visit a specific restaurant [6].

Fifth, this study discovered a significant influence of brand social media visual communication on customer visit intentions. The finding implies that how restaurants interact on social media platforms significantly influences consumers’ decisions to dine at their venues. Prior research echoed the findings, demonstrating that effective social media communication is critical in changing customer attitudes and intentions. It emphasizes the need for restaurants to build effective and engaging social media marketing strategies to retain existing and attract new customers [22,42]. Customers may perceive a brand’s social media visual communication based on their motivation level, which may influence their decisions [27,29]. Further, the findings revealed that customer demographics such as gender, age, and income had no influence on customer visit intentions. Therefore, future research may consider customer demographics in similar studies to validate the findings.

Sixth, the argument quality of online reviews as perceived informativeness acted as a decisive mediator between the relationships between CGPs and perceived persuasiveness and between CGPs and customer visit intentions. Likewise, the argument quality of online reviews as perceived persuasiveness was a critical mediator between the relationships between CGPs and perceived informativeness and between CGPs and customer visit intentions. The argument quality of online reviews (perceived informativeness and persuasiveness) is essential for studying the underlying mechanisms influencing customers’ behavioral intentions and driving their decisions [17]. Finally, the findings revealed that the interplay between brand social media visual communication and customer-generated photos did not influence customer intentions to visit the restaurant. The findings deviate from the previous research, indicating that brand and consumer-generated content on social media enhance consumer engagement and behavioral intentions [2,49]. The non-significant interaction effects can be linked to cultural and technological factors because many Pakistani customers prefer positive WOM and recommendations from family and friends [57]. In this context, brand social media visual communication and customer-generated photos may be ineffective. In addition, the non-significant interaction effect may emphasize the dynamic nature of customer behavior and the need for continuously monitoring and adapting marketing strategies [22]. Future research may pursue this line of action to validate the research findings. Restaurant marketers should continuously evaluate market trends, customer preferences, and social media dynamics to fine-tune their strategy and ensure effectiveness in driving customer intentions to visit the restaurant.

### 5.1. Theoretical contributions

This research contributes novel insights to HSM and ELM in various aspects. First, this research validates the scales in the restaurant context of the hospitality industry. Second, this research contributes to the HSM and ELM by demonstrating the customer-generated photos (CGPs)’ considerable effects on the argument quality of online reviews, such as perceived informativeness and persuasiveness. The HSM indicates that people process information heuristically (using mental cues) and systematically (via rigorous evaluation of arguments) [25]. Thus, CGPs serve as heuristic and systematic indicators, impacting how customers perceive online reviews. According to the ELM perspective, customer motivation defines the central and peripheral routes for information processing, which assist customers to define their informativeness and persuasiveness [27]. Thus, CGPs can delineate customers’ motivation, which can improve their level of informativeness and persuasiveness. Third, CGPs are powerful communication tools that can serve as persuasive visual messages, conveying information [7] about the restaurant’s menu offerings, atmosphere, and overall experience. The ELM suggests that individuals apply the central and peripheral routes to process information based on their motivation, which can influence their attitudes and behavioral intentions [28]. CGPs may serve as the central or peripheral paths based on customers’ motivation, which can affect their behavioral intentions. Fourth, the HSM demonstrates that when people participate in systematic processing, they meticulously assess the argument quality of online reviews [17]. Higher perceived informativeness and persuasiveness are more likely to result in positive customer intentions to visit the restaurant.

Fifth, the HSM states that people frequently use heuristic and systematic clues [17,25], such as social media presence and its valuable reviews, to quickly shape their intentions. Likewise, customers perceive social media posts as important information about the brand’s offerings, promotions, or atmosphere, influencing their decision to visit the restaurant. Sixth, the HSM delineates that the argument quality of online reviews, comprising perceived informativeness and persuasiveness, offers a systematic process of information that assists individuals in shaping their behavioral intentions [17]. Thus, customers who are motivated and able to process information thoroughly participate in systematic processing. High-quality online reviews can provide in-depth information about the restaurant’s attributes and, through a systematic process, influence customer decisions. Finally, brand social media visual communication can be a source of motivation for getting deep or affective information based on the motivation of customers that can influence their behaviors [29].

### 5.2. Managerial contributions

This research, particularly the IPMA, assists restaurant marketing managers in enhancing their strategic decision-making skills in various aspects. First, considering the positive impacts of customer-generated photos on the components of argument quality of online reviews including perceived informativeness and persuasiveness, and customer visit intentions to the restaurants, customers motivation can be enhanced by providing good quality of food, ambient environment, and competitive prices, which may compel them to share food photos on social media platforms like Facebook and Instagram. These photos can motivate other customers to improve their information and persuasion, as well as their visit intentions. Second, using the components of argument quality of online reviews, including perceived informativeness and persuasiveness, managers can improve customer visit intentions to the restaurants. The online reviews can be enhanced through virtual and physical provision of services to customers. Third, managers should encourage customers to provide high-quality reviews by offering discounts or freebies for detailed feedback on social media platforms. Likewise, the managers should highlight persuasive reviews on social media platforms by describing food quality, dining experience, and customer service to enhance customer visit intentions.

Fourth, managers should use a robust social media visual communication strategy, including appealing customer-shared food pictures, behind-the-scenes glimpses, and customer testimonials. It can help other customers process brand messaging heuristically. Brand visual information must be consistent across social media platforms to enhance customer interactions, which may positively influence customer intentions. Finally, managers may segment customers considering the favorable mediating effects of perceived informativeness and persuasiveness. For example, highly motivated customers may use central routes for deep information processing, whereas less motivated customers can use peripheral routes for information processing, which may influence their attitudes and behavioral intentions.

### 5.3. Limitations and future research agenda

This study is not devoid of limitations. First, data was collected from customers who looked at photos of various cuisines and restaurants. Future research might narrowly focus on a specific segment of restaurants to elucidate insights specific to that segment. Moreover, future research could explore the role of these various restaurant types as moderators, uncovering novel insights. Second, this study was conducted exclusively within a single South Asian country, Pakistan. Future research may seek to widen their reach by investigating other regions within Asia or undertaking cross-regional comparisons between Asia and Europe to improve the generalizability of findings. Third, this study employed a snowball sampling methodology. Future research could adopt a random sampling methodology to enhance the generalizability of findings. Fourth, this study was based on customers’ perceptions. Future research may integrate content analysis and machine learning to reveal more insights. Fifth, this study is limited to customer behavioral intentions. Further research can investigate other variables, such as customer loyalty, customer repeat visit intentions, as well as customer emotions. Sixth, this study was conducted in the hospitality industry, particularly focusing on restaurants. The findings may have relevance in other contexts, such as the retail and apparel industries. Finally, this study used a moderator from the restaurant perspective. Future studies might incorporate additional moderators from the customers’ perspectives, such as customer engagement, involvement, and emotional responses.

## Supporting information

S1 Data Data Analysis File. (CSV)
